# Supplementary material for: The B-cell inhibitory receptor CD22 is a major factor in host resistance to Streptococcus pneumoniae infection
Source: PLoS Pathog. 2020 Apr 23;16(4):e1008464. doi: 10.1371/journal.ppat.1008464 (PMC7179836; doi:10.1371/journal.ppat.1008464)
Supplement: S1 Fig — The SNP found in the cd22 gene in the CBA/CaOlaHsd inbred strain is located at position 7:30,877,586 (chromosome position highlighted in light blue), and is absent in all other sequenced strains. The SNPs in the figure have the following consequences: T (dark red) stop gained; A, C, G and T (light red), NMD transcript variant; A, G and T, (yellow) missense/initiator codon variant; A and C (green), synonymous/stop retained variant. The asterisk symbol on the SNP indicate that the SNP was observed in multiple sequences. Exon 4 SNPs (blue highlighted box with an arrow indicating the reverse orientation) are located between SNP positions 7:30,877,522–30,877,839. Data were collected from the Wellcome Trust’s Sanger Institute website (http://www.sanger.ac.uk). (PDF) [file ppat.1008464.s001.pdf]

Exon 4 → (rows 30,877,522 to 30,877,839)

Gene	Chromosome	Position	dbSNP	Reference	CBA/CaOlaHsd	129P2/OlaHsd	129S1/SvImJ	129S5SvEvBrd	AKR/J	A/J	BALB/cJ	BTBR T+ Itpr3tf/J	BUB/BnJ	C3H/HEH	C3H/HeJ	C57BL/10J	C57BL/6NJ	C57BR/cdJ	C57L/J	C58/J	CAST/EiJ	CBA/J	DBA/1J	DBA/2J	FVB/NJ	I/LnJ	KK/HiJ	LEWES/EiJ	LP/J	MOLF/EiJ	NOD/ShiLtJ	NZB/B1NJ	NZO/HlLtJ	NZW/LacJ	PWK/PhJ	RF/J	SEA/GnJ	SPRET/EiJ	ST/bJ	WSB/EiJ	ZALENDE/EiJ
Cd22	7	30,876,625	rs45776325	G	C*	A*	A*	A*	A*	A*	A*	A*	-	-	-	-	-	A*	A*	A*	A*	-	A*	A*	-	A*	-	A*	A*	A*	A*	A*	A*	A*	A*	A*	A*	A*	A*	A*	A*
Cd22	7	30,876,670	rs50541180	A	-	-	-	-	G*	-	-	G*	-	-	-	-	-	-	-	-	-	-	G*	G*	-	-	-	-	G*	G*	-	G*	G*	G*	-	-	G*	-	-	G*	-
Cd22	7	30,876,821	rs49113036	G	-	-	-	-	C*	-	-	C*	-	-	-	-	-	-	-	-	C*	-	C*	C*	-	-	-	-	C*	C*	-	C*	C*	C*	-	-	C*	-	-	C*	-
Cd22	7	30,876,826	rs262991762	C	-	-	-	-	-	-	-	-	-	-	-	-	-	-	-	-	-	-	T*	T*	-	-	-	-	-	T*	-	T*	T*	T*	-	-	-	-	-	-	-
Cd22	7	30,876,923	rs225055652	A	-	-	-	-	-	-	-	-	-	-	-	-	-	-	-	-	-	-	T*	T*	-	-	-	-	-	-	-	T*	T*	T*	-	-	-	-	-	-	-
Cd22	7	30,877,115	rs51188576	G	-	-	-	-	A*	-	-	A*	-	-	-	-	-	-	-	-	A*	-	A*	A*	-	-	-	-	A*	A*	-	A*	A*	A*	-	-	A*	-	-	A*	-
Cd22	7	30,877,157	rs45818091	T	-	-	-	-	-	-	-	-	-	-	-	-	-	-	-	-	-	-	C*	C*	-	-	-	-	-	C*	-	C*	C*	C*	-	-	-	-	-	-	-
Cd22	7	30,877,232	rs8240970	C	-	-	-	-	T*	-	-	T*	-	-	-	-	-	-	-	-	-	-	-	-	-	-	-	-	T*	-	-	-	-	-	-	-	T*	-	-	-	-
Cd22	7	30,877,335	-	C	-	-	-	-	-	-	-	-	-	-	-	-	-	-	-	-	-	-	-	-	-	A*	-	-	-	-	-	-	-	-	-	-	-	-	A*	-	-
Cd22	7	30,877,406	rs8240969	G	-	-	-	-	-	-	-	-	-	-	-	-	-	-	-	-	-	-	-	-	-	-	-	-	-	-	-	-	-	-	-	-	-	A*	-	-	-
Cd22	7	30,877,522	rs8240968	C	-	-	-	-	T*	-	-	T*	-	-	-	-	-	-	-	-	-	-	-	-	-	-	-	-	T*	-	-	-	-	-	-	-	T*	-	-	T*	-
Cd22	7	30,877,528	rs8240967	G	-	-	-	-	A*	-	-	A*	-	-	-	-	-	-	-	-	-	-	-	-	-	-	-	-	A*	-	-	-	-	-	-	-	A*	-	-	A*	-
Cd22	7	30,877,557	rs8240966	T	-	-	-	-	-	-	-	-	-	-	-	-	-	-	-	-	-	-	A*	A*	-	-	-	-	-	A*	-	A*	A*	A*	-	-	-	-	-	-	-
Cd22	7	30,877,583	rs8240965	C	-	-	-	-	-	-	-	-	-	-	-	-	-	-	-	-	-	-	T*	T*	-	-	-	-	-	T*	-	T*	T*	T*	-	-	-	-	-	-	-
Cd22	7	30,877,586	-	C	T*	-	-	-	-	-	-	-	-	-	-	-	-	-	-	-	-	-	-	-	-	-	-	-	-	-	-	-	-	-	-	-	-	-	-	-	-
Cd22	7	30,877,592	rs8240964	A	-	-	-	-	-	-	-	-	-	-	-	-	-	-	-	-	-	-	G*	G*	-	-	-	-	-	G*	-	G*	G*	G*	-	-	-	-	-	-	-
Cd22	7	30,877,594	rs8240963	A	-	-	-	-	-	-	-	-	-	-	-	-	-	-	-	-	-	-	G*	G*	-	-	-	-	-	G*	-	G*	G*	G*	-	-	-	-	-	-	-
Cd22	7	30,877,607	rs8240962	A	-	-	-	-	-	-	-	-	-	-	-	-	-	-	-	-	-	-	G*	G*	-	-	-	-	-	G*	-	G*	G*	G*	-	-	-	-	-	-	-
Cd22	7	30,877,654	rs8240957	G	-	-	-	-	T*	-	-	T*	-	-	-	-	-	-	-	-	-	-	T*	T*	-	-	-	-	T*	T*	-	T*	T*	T*	-	-	T*	-	-	T*	-
Cd22	7	30,877,656	rs8240956	G	-	-	-	-	A*	-	-	A*	-	-	-	-	-	-	-	-	-	-	-	-	-	-	-	-	A*	-	-	-	-	-	-	-	A*	-	-	A*	-
Cd22	7	30,877,795	rs215759590	T	-	-	-	-	-	-	-	-	-	-	-	-	-	-	-	-	-	-	-	-	-	-	-	-	-	-	-	-	-	-	-	-	-	C*	-	-	-
Cd22	7	30,877,825	rs47811910	C	-	-	-	-	-	-	-	-	-	-	-	-	-	-	-	-	-	-	T*	T*	-	-	-	-	-	T*	-	T*	T*	T*	T*	-	-	T*	-	-	T*
Cd22	7	30,877,837	rs50937650	A	-	-	-	-	-	-	-	-	-	-	-	-	-	-	-	-	G*	-	G*	G*	-	-	-	-	-	G*	-	G*	G*	G*	G*	-	-	G*	-	-	-
Cd22	7	30,877,839	rs3698643	G	C*	A*	A*	A*	-	A*	A*	-	-	-	-	-	-	A*	A*	A*	-	-	-	-	-	A*	-	A*	-	-	A*	-	-	-	-	A*	-	-	A*	-	A*
Cd22	7	30,877,931	rs237327268	C	-	-	-	-	-	-	-	-	-	~	-	-	-	-	-	-	-	-	-	-	-	-	-	-	-	-	-	-	-	-	-	-	-	T*	-	-	-
Cd22	7	30,877,956	rs248162986	T	-	-	-	-	-	-	-	-	-	-	-	-	-	-	-	-	C*	-	C*	C*	-	-	-	-	-	C*	-	C*	C*	C*	-	-	-	-	-	-	-

S1 Fig. The stop codon identified in the *cd22* gene of CBA/Ca does not occur in 37 inbred mouse strains from Jackson Laboratories. The SNP found in the *cd22* gene in the CBA/CaOlaHsd inbred strain is located at position 7:30,877,586 (chromosome position highlighted in light blue), and is absent in all other sequenced strains. The SNPs in the figure have the following consequences: T (dark red), stop gained; A, C, G and T (light red), NMD transcript variant; A, G and T (yellow), missense/initiator codon variant; A and C (green), synonymous/stop retained variant. The asterisk symbol on the SNP indicate that the SNP was observed in multiple sequences. Exon 4 SNPs (blue highlighted box with an arrow indicating the reverse orientation) are located between SNP positions 7:30,877,522-30,877,839. Data were collected from the Wellcome Trust's Sanger Institute website (http://www.sanger.ac.uk)
