# Supplementary material for: The B-cell inhibitory receptor CD22 is a major factor in host resistance to Streptococcus pneumoniae infection
Source: PLoS Pathog. 2020 Apr 23;16(4):e1008464. doi: 10.1371/journal.ppat.1008464 (PMC7179836; doi:10.1371/journal.ppat.1008464)
Supplement: S2 Fig — Flow cytometric analysis of splenocytes and lung cells from BALB/c and CBA/Ca mice stained for CD19 and CD22 (MAb Cy34.1) 24 h after intranasal infection with S. pneumoniae. (PDF) [file ppat.1008464.s002.pdf]

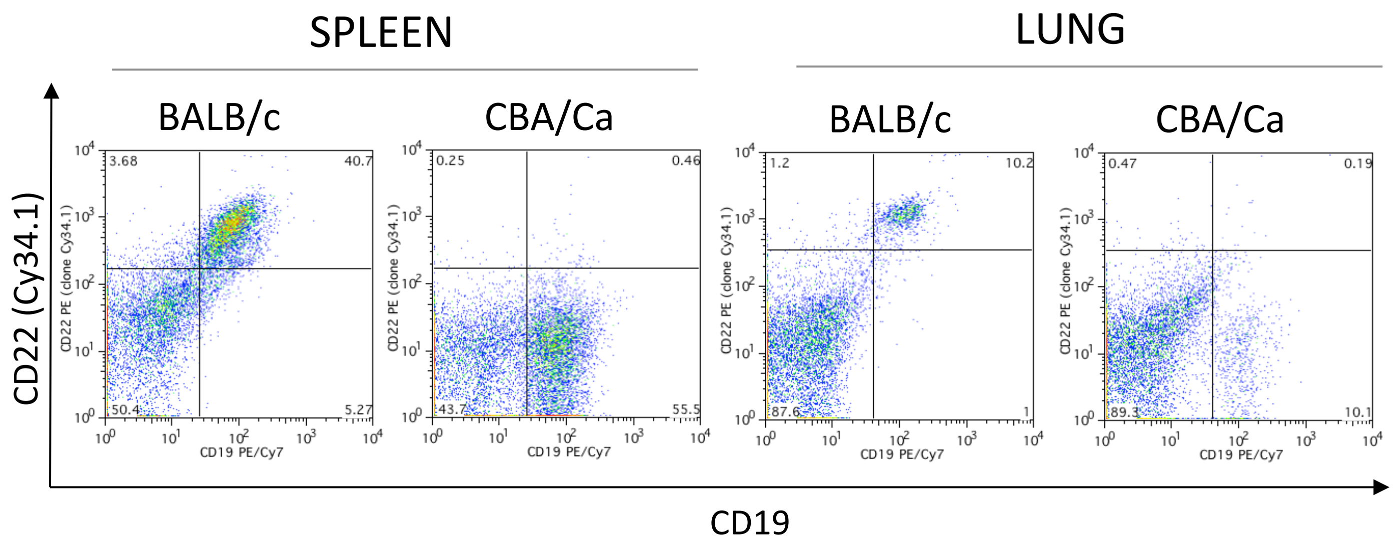

<u>S2 Fig</u>. B cells in CBA/Ca mice do not express CD22 as revealed by staining with Cy34.1 antibody. Flow cytometric analysis of splenocytes and lung cells from BALB/c and CBA/Ca mice stained for CD19 and CD22 (MAb Cy34.1) 24 h after intranasal infection with *S,pneumoniae.*
