# Supplementary material for: The B-cell inhibitory receptor CD22 is a major factor in host resistance to Streptococcus pneumoniae infection
Source: PLoS Pathog. 2020 Apr 23;16(4):e1008464. doi: 10.1371/journal.ppat.1008464 (PMC7179836; doi:10.1371/journal.ppat.1008464)
Supplement: S3 Fig — Mice were intranasally infected with 1 x 10^6^ CFU and organs were collected at 0, 6, 12 and 24 hours post-infection. B cell numbers were determined by gating IgM-/+ CD19+ cells in lung (A) and spleen (B) by flow cytometry. Significant increase of B cells numbers over time (p<0.01, from 0 to 24 hours p.i.) in the lung tissue of BALB/c mice (Figure A) and significant decrease in the spleen of BALB/c mice (p<0.01). Examples of the 24 hours time point are shown in the top panels. Data are representative of two independent experiments with > 4 mice per group. The p-values (** = p<0.01) were obtained with two-way ANOVA. (PDF) [file ppat.1008464.s003.pdf]

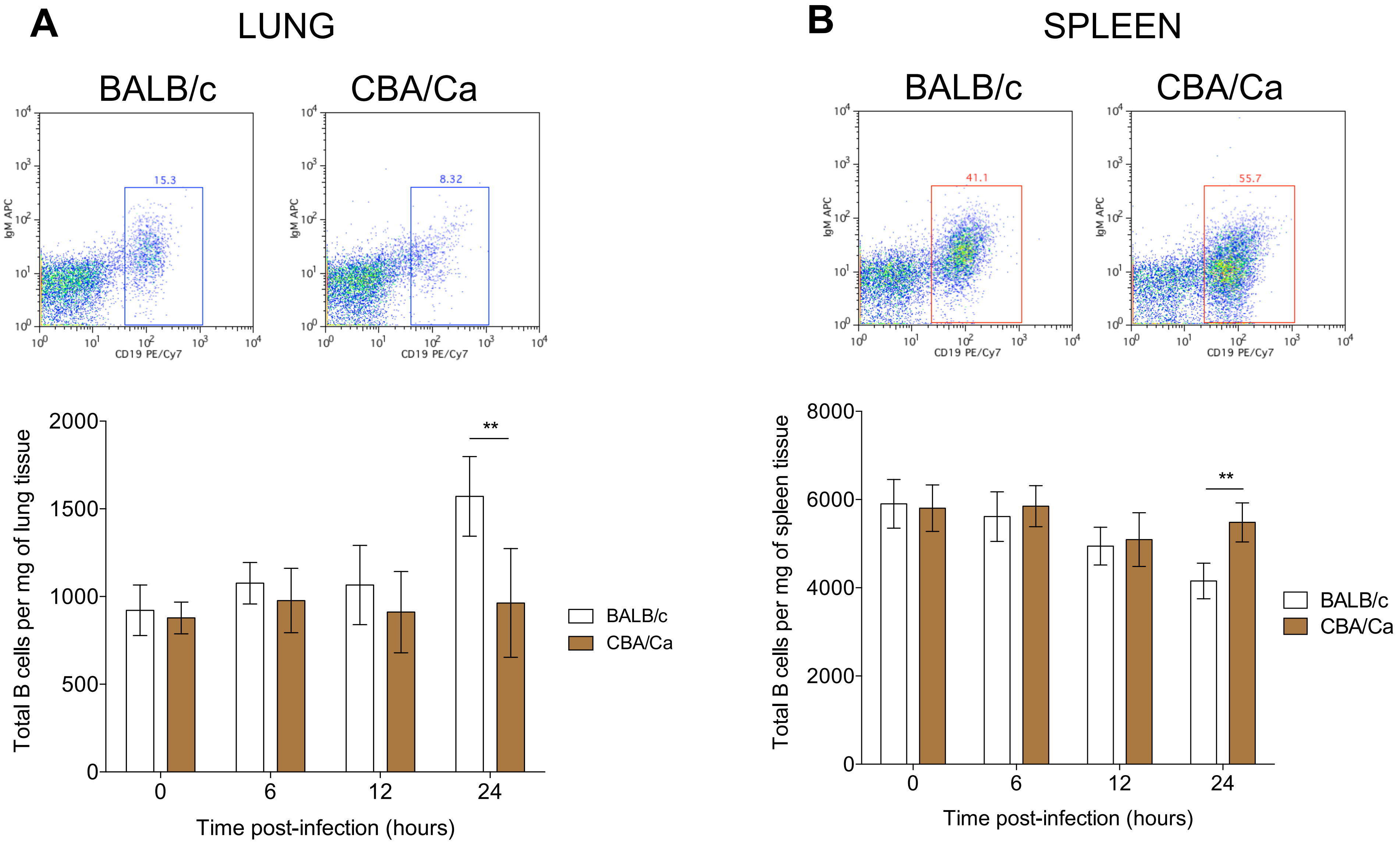

S3 Fig Impaired B cell infiltration of B cell into the lung in CBA/Ca mice after pneumococcal infection.
Mice were intranasally infected with 10^6 CFU and organs were collected at 0, 6, 12 and 24 hours post-infection.
B cell numbers were determined by flow cytometric analysis in lung (A) and spleen (B). Significant increase of B cells numbers over time (** $p < 0.01$, from 0 to 24 hours p.i.) in the lung tissue of BALB/c mice (Figure A) and significant decrease in the spleen of BALB/c mice (** $p < 0.01$). Examples of the 24 hour time points are shown in the top panels. Data are representative of two independent experiments with > 4 mice per group. ** $p < 0.01$ (two-way ANOVA).
