# Supplementary material for: The B-cell inhibitory receptor CD22 is a major factor in host resistance to Streptococcus pneumoniae infection
Source: PLoS Pathog. 2020 Apr 23;16(4):e1008464. doi: 10.1371/journal.ppat.1008464 (PMC7179836; doi:10.1371/journal.ppat.1008464)
Supplement: S4 Fig — Mice were intranasally infected with 106 CFU and BAL fluid was collected at 0, 6, 12 and 24 hours post-infection. Total IgM and IgG levels were determined by ELISA. Data are representative of two independent experiments with > 4 mice per group. ** p< 0.01 (two-way ANOVA). (PDF) [file ppat.1008464.s004.pdf]

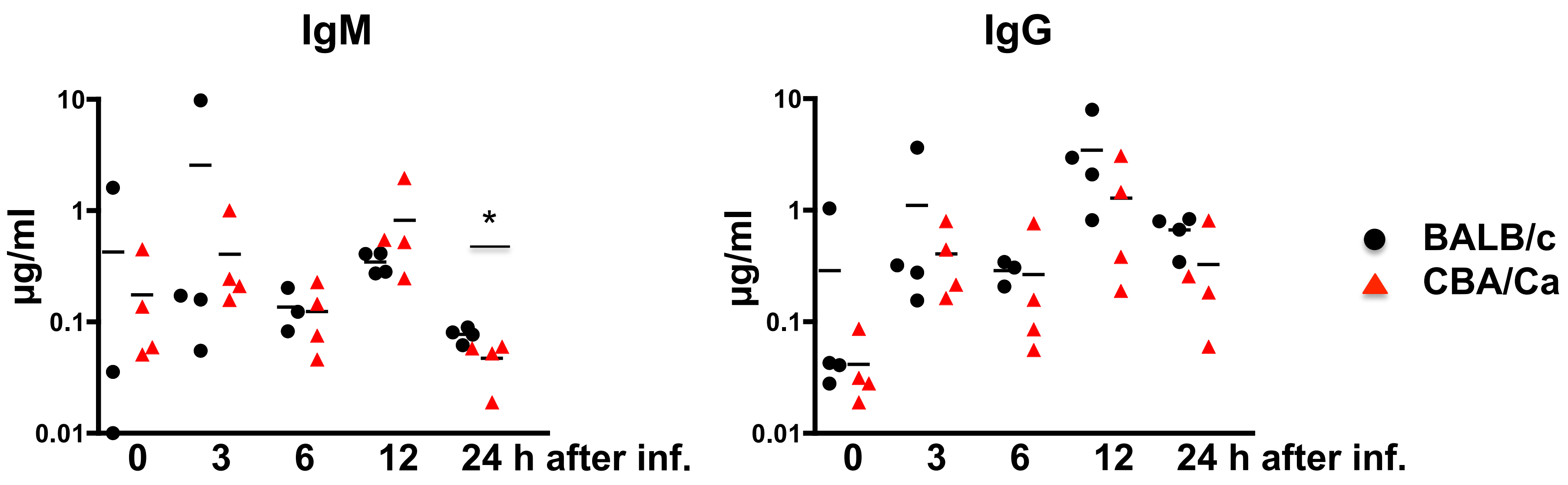

S4 Fig Slighly impaired IgM levels at 24 hours in BAL fluid of CBA/Ca mice after pneumococcal infection.
Mice were intranasally infected with 10^6 CFU and BAL fluid was collected at 0, 6, 12 and 24 hours post-infection.
Total IgM and IgG levels were determined by ELISA. Data are representative of two independent experiments with > 4 mice per group.
** $p< 0.01$ (two-way ANOVA).
