# Supplementary material for: The B-cell inhibitory receptor CD22 is a major factor in host resistance to Streptococcus pneumoniae infection
Source: PLoS Pathog. 2020 Apr 23;16(4):e1008464. doi: 10.1371/journal.ppat.1008464 (PMC7179836; doi:10.1371/journal.ppat.1008464)
Supplement: Attachment — Submitted filename: point by point response2.pdf [file ppat.1008464.s005.pdf]

Point-by-point response

Comment by the editors:

The reviewers were overall positive about the findings. One important aspect that should be addressed is to provide more convincing evidence that it is indeed B cell recruitment rather than in situ proliferation that is responsible for the phenotype. Moreover, the manuscript would be strengthened with data showing that in the pneumococcal model, there are also fewer IRA B cells (rather than just with the E coli infection).

Response:
We performed two additional experiments that addressed these points:

1) An adoptive transfer experiment that addressed the question whether it is defective B cell recruitment or impaired proliferation in situ in the lung.

2) An additional pneumococcal infection to show that also after this infection, IRA B cells are reduced in the lung.

See responses to the reviewers for details.

The editors also asked us to avoid any statements of “data not shown” in the manuscript. Therefore we have included those data, which were previously not shown, as new Suppl. Fig.2 and Fig.4 panels G,H, respectively.

Reviewer #1 (summary): The authors present convincing evidence that a SNP resulting in loss of function of CD22 largely accounts for the increased susceptibility of CBA/Ca mice to pneumococcal lung infection, relative to other inbred mouse strains. Moreover, CD22-/- mice in the C57BL/6 background exhibited enhances susceptibility relative to the wild type. Clues as to the underlying mechanism were also provided by data showing a defect in recruitment of IRA B cells, resulting in lower levels of GM-CSF and IgM. This study provides new information on the hitherto unrecognised role of CD22 in host resistance to bacterial infection and paves the way for further molecular epidemiological/GWAS studies, including in human populations. For this reason, it is an important contribution.

Response
Thank you very much for this positive evaluation.

Reviewer #1: (major issues) Page 8. Whilst the difference between C57BL/6 and CD22-/- mice in numbers IRA B cells in lungs after challenge with E. coli are convincing, it is a little puzzling why the authors did not attempt the same experiments in mice challenged with D39. It would be preferable if such data were included in the manuscript.

Response
We agree and have now performed such an experiment. It is included as new Fig.7. Indeed, we find the same reduction of IRA B cells in the lung of CD22-/- mice, also after pneumococcal infection. It is a very similar finding compared to the E.coli infection.

Reviewer #1: (minor issues) Line 84. pneumoniae.

Line 92. LOD needs to be defined.

Line 93. The definition of QTL needs to be here rather than on line 99.

Line 176. It is not strictly correct to say there were no bacteria in the blood of infected C57BL/6 mice, as there was 1 culture-positive mouse at 12 h.

Line 187. should be Suppl Fig 2B.

Line 211. GM-CSF in blood was actually significantly higher in CD22-/- mice at 6 h, so perhaps this sentence needs to be qualified.

Fig. 4. The A, B, C and D labels are missing from the panels.

Response
All these mistakes have been corrected. Thank you.

Reviewer #2 (summary): In this manuscript, the authors describe an important and previously unrecognized role for CD22 in susceptibility of infection to Streptococcus pneumoniae. This works stems from an earlier report by the authors on a loci within CBA/Ca mice that causes high susceptibility to infection. This loci contains CD22, an important B cell inhibitory co-receptor, and it is found that a premature stop codon is located in the Cd22 gene within this strain that causes no functional CD22 to be expressed on their surface. To test if this loss of functional CD22 in CBA/Ca mice is responsible, the author carry out studies in C57BL/6 mice on a CD22+/+ and CD22-/- background. Studies in C57BL/6 mice support the authors' claim that CD22 is directly involved in susceptibility Streptococcus pneumoniae infection. The authors search for mechanism is far from complete, but they do reveal a key differences in B cell recruitment to the lung, supporting earlier reports of B cells being an important immune cell limiting Streptococcus pneumoniae infection in mice. The results are interesting, with many key questions remaining, but should be published after the points below are addressed:

Response
Thank you for this overall positive evaluation.

Reviewer #2 (minor issues): 1. No direct evidence is provided to demonstrate a defect in B cell recruitment or strictly that it is CD22 expression on B cells that is the key factor. Accordingly, I have 4 inquiries:

a) Can the authors rule out impaired proliferation of CD22-/- resident lung B cells?

b) Is it known that B cells home to the lung in a similar manner as the Peyer's patches? Are HEVs involved? If so, it would certainly be worth discussing this similarity more directly. If not, it should be stated that a previously undiscovered homing mechanism would necessarily need to be at play.

Response
We felt that this is an important question. Therefore we did additional experiments: we performed adoptive transfer experiments of mixed labelled WT and CD22-/- splenocytes into WT recipients and examined homing to the lung and spleen. We observed no difference in short term homing to the lung (2 h after i.v. injection), but after 48 hours, when less CD22-/- B cells were found in the lung (new Fig. 5). We interpret the data as follows: it is not so much the defective homing to the lung that is the mechanism, but rather the impaired proliferation in situ or the impaired survival. The latter two we cannot distinguish. According to the new data, we deleted the previous discussion about a homing mechanism which could be similar to Peyer's patch homing and discuss impaired proliferation/ and or survival as a mechanism (both in the results section, as well as in discussion). We feel that these new data are important towards explaining the mechanism of the findings.

c) Can the authors rule out a role for DCs given that CD22 is known to be expressed on these cell types? Is there any known link between DCs and S. pneumoniae infection? In this respect, it is worth pointing out that Ma et al. (2012, J Virol) determined that loss of CD22 results in a significant decrease in several cytokines which was due to loss of CD22 in cDC2.

Response
We cannot rule out DCs, however DCs are not a major cellular population recruited to the lung after pneumococcal infections. We analysed the major populations of cells infiltrating the lung after these infections, i.e. neutrophils, macrophages, eosinophils, B- and T-cells. Besides CD22 expression on DC subpopulations is rather weak.

d) Does S. pneumoniae express ligands of CD22 that could be contributing to these effects? Have the authors (or anyone previously) looked at staining of S. pneumoniae with CD22-Fc?

Response
There is no indication in the literature that S. pneumonia does express sialic acids. CD22 is highly specific for the Neu5Ac-2,6-Gal linkage. Bacteria usually not have these ligands. Some bacteria steel sialic acids from the host (Neisseria, Haemophilus influenza, Group B streptococcus), but this is not known for S. pneumonia.

2. For the study in Ref19, it would be worth discussing any possible differences in: a) route of administration, b) strain background, c) primary site of infectivity between Staphylococcal and Streptococcal infection between studies.

Response
I added the information in the discussion. It was the same strain of mice, but the infection was done i.v. and could therefore have a completely different outcome.

3. Please provide suitable ref for the statement on Lines 146-148 about antibody specificity.

Response
Two references were added for the antibodies

4. Lines 224-226: Data not shown?

5. Lines 228-237: Assume this is referring to intranasal infection? Since the experiment described directly above is intratracheal, it should be clearly stated.

Response
Yes, both refers to intratracheal infections in the experiment, this is now clearly stated.

6. Are there any reported SNPs in humans that result in loss of CD22 protein or loss of functional CD22? Worth mentioning either way in the discussion.

Response
Such SNPs have not been described so far. Is now added to the discussion.

7. Typos:
Line 222: Type: C22
Line171: CD22 -/- to CD22-/-

Response
these were corrected. Thank you.

Reviewer #3 (summary): The paper by Fernandes et al., seeks to describe the role of CD22 in susceptibility to pneumococcal disease. The authors hypothesis is that a point mutation in CD22 in CBA mice is the cause of their susceptibility to pneumococcal disease. They base their argument on

the fact that CD22 KO mice are also susceptible to pneumococcal infection. This is an interesting hypothesis, however the main issue is that the data supporting this comes from the CD22 KO which is in the C57 background. Therefore, it is difficult to see how the authors can prove that CD22 really is the factor leading to susceptibility in CBA mice. Yes, there is an association i.e. it seems that in C57 mice lack of CD22 does lead to increased susceptibility to pneumococcal infection, but we need to see a direct link/proof in CBA mice. This is not provided, which is surprising as there are a number of experiments which could have been done to address this.

The only data provided from the CBA mouse background is the supplementary data which shows total B cells in lung and spleen tissue from CBA vs Balb/c mice. What is interesting is that there are no differences in B cells between these two strains of mice in lung or spleen, with the exception of 24hrs in lung B cells in CBA mice, but by this time the CBA mice already have high CFU levels in their blood based on previous papers and are dying from their infection. Indeed, CBA mice develop bacteremia as early as 6hrs post infection, hence the essential role of B cells in this mouse strain is not really supported.

See comments in additional exps that may strenghten paper.

Response
The reviewer asks for more evidence that the phenotypes of the two mouse pairs (CBA/CA versus BALB/c and CD22-/- versus C57BL/6) is the same and whether defective B cell populations and defective antibody responses are also involved in CBA/Ca mice. We have decided to include the survival data, disease scores and bacteremia of CBA/Ca mice (new Fig.1) in order to make the comparison to CD22-/- on the B6 background (new Fig.3) better comparable. The phenotype looks in all aspects quite similar, but with a bit stronger severity in CBA/Ca mice. After finding the mutation in *Cd22* in CBA/Ca, we concentrated on analysis of the CD22-/-(B6) vs. B6 WT mice, as they have the same genetic background and therefore are directly comparable, whereas there may be several aspects that influence B cell numbers or antibody levels in the lungs of mice with totally different genetic backgrounds. See also the more specific answers below.

Reviewer #3 (major issues): [1] It would be good to see IgM and IgG levels (including subsets) in CBA vs Balb/c mice (lungs and blood) following infection with pneumococci at the timepoints used (0, 6, 12, 24 etc).

Response
We have included the IgM and IgG analysis of BAL of CBA/Ca vs BALB/c mice after pneumococcal infections. We find only mild differences (lower IgM at 24h). Now included as new Suppl. Fig.3.

[2] It would be good to see a more detailed B cell analysis, including subsets (B1, B2 etc) in above groups/times in lungs.

Response
We have decided to do the detailed cellular analysis on the B6 background, as this is the same genetic background as the CD22-deficient mice, as we thought this is more important to do.

[3] Would be interesting to see adoptive transfer of B1 cells into CBA mice. If their susceptibility is due to a lack of these cells, then adoptive transfer would answer the question of whether this is true or not.

Response
This cannot be done easily, as the experiment would have to involve transfer of WT B1 cells (i.e. from BALB/c mice) into CBA/Ca. This would lead to rejection due to different genetic background. However, we have done adoptive transfer experiments to look for homing and survival of CD22-/- vs. WT B cells in the lung (new Fig.5, see also the response to reviewer 2). Thank you for your comments.
