# Supplementary material for: The B-cell inhibitory receptor CD22 is a major factor in host resistance to Streptococcus pneumoniae infection
Source: PLoS Pathog. 2020 Apr 23;16(4):e1008464. doi: 10.1371/journal.ppat.1008464 (PMC7179836; doi:10.1371/journal.ppat.1008464)
Supplement: Attachment — Submitted filename: Point-by-point 2nd rev.pdf [file ppat.1008464.s006.pdf]

Point-by-point response for 2nd revision

Reviewer #2:

1) It would strengthen the presentation to show individual data points in the bar graphs.

We have done this for all figures containing bar graphs, i.e. Fig. 4, 5, 6, 7 and 8.

2) i) In the new adoptive transfer data, the authors describe it as 'migration'. While I am admittedly not an expect in this area, I think migration refers more to movement towards a signal, which may be more appropriate during infection. Since these particular experiments were carried out in healthy mice, I believe homing is the more correct term.

We agree and have changed migration into homing throughout the manuscript (lines 211, 213, 321).

ii) They describe infer that the adoptive transfer experiment provides insight into 'proliferation in the lung'. I do not see evidence that they examined this aspect (which could have been done in the context of fluorescence dilution). It appears that they have only investigated homing and retention. Wording to describe this in the text should be modified appropriately.

We analysed the CTV and CFSE dilution of the two B cell populations after cellular transfer and did not see differences between WT and CD22-/- B cells. This is now mentioned in the text and the paragraph is re-written (lines 224-227)

3) lines 46 and 113- it is written 'a lack of B cells' in CD22-null mice post infection. While B cell population in the lungs CBA/Ca mice is significantly reduced after bacterial inoculation, there are a few that were observed. I think it is an overstatement to say that these mice lack the B cells.

We changed this to "strongly reduced B cells".

4) In Figures 7B/7C and 8C/8D, the authors should report if these are normalized (e.g. per mg tissue).

This information was now added to the figure legends (lines 714 and 726)
